# A survey of monitoring tap water hardness in Japan and its distribution patterns

**DOI:** 10.1038/s41598-021-92949-8

**Published:** 2021-06-29

**Authors:** Mayumi Hori, Katsumi Shozugawa, Kenji Sugimori, Yuichiro Watanabe

**Affiliations:** 1 Komaba Organization for Educational Excellence, The University of Tokyo, 3-8-1 Komaba, Meguro, Tokyo 153-8902 Japan; 2 Department of General Systems Studies, Graduate School of Arts and Sciences, The University of Tokyo, 3-8-1 Komaba, Meguro, Tokyo 153-8902 Japan; 3 Department of Biology, School of Medicine, Faculty of Medicine, Toho University, 5-21-16, Omori-Nishi, Ota-ku, Tokyo 143-8540 Japan; 4 Department of Life Sciences, Graduate School of Arts and Sciences, The University of Tokyo, 3-8-1 Komaba, Meguro, Tokyo 153-8902 Japan; 5 Integrated Human Sciences Program for Cultural Diversity, The University of Tokyo, 3-8-1 Komaba, Meguro, Tokyo 153-8902 Japan; 6 Graduate Program on Environmental Sciences, The University of Tokyo, 3-8-1 Komaba, Meguro, Tokyo 153-8902 Japan

**Keywords:** Hydrology, Environmental sciences, Environmental chemistry, Environmental monitoring

## Abstract

We conducted a comprehensive overall tap water hardness assessment for Japan. Tap water was collected from 665 points throughout Japan, and its standing position was quantitatively clarified by prefecture. The mean and median hardness of tap water in Japan was 48.9 ± 25.8 (1σ SD) and 46.0 mg/L, respectively. Compared with 27 other countries, Japan exhibited soft water with low-mineral content. Water hardness tended to be high in the Kanto region and low in the Hokkaido and Tohoku regions. The impact of the distribution system’s water pipes on tap water hardness is discussed using a unified index to evaluate variations in hardness from raw to tap water. A comparison of the variations in hardness showed that hardness variations from raw to purified water and from purified to tap water exhibited a 20% variation range. Furthermore, tap water hardness and its fluctuations in any region of Japan were found to be caused by raw water hardness. It was demonstrated that the distribution pipe system had no large impacts on water hardness.

## Introduction

Tap water is an essential resource that people use every day, not only for daily life, such as drinking and cooking, but also for industrial contexts. In recent years, however, due to dissatisfaction with the taste of tap water and concerns about its safety, it is getting popular for citizens to purchase mineral water instead of tap water to drink^1^. In a summary of the Mineral Water Association of Japan, the mineral water consumption per capita in Japan in 2019 was 31.7 L/year/person, which was much less than in Western countries (e.g., [unit: L/year/person] in the United States [US]: 119.0, Canada: 58.0, United Kingdom: 38.0, Germany: 125.7, France: 147.4, Switzerland: 102.7)^2^. Over the past 15 years, however, the consumption of bottled mineral water increased in Japan to 14.4 L/person/year in 2005 and 19.6 L/person/year in 2007. Then, it did not increase (19.7 L/year/person in 2010) until 2011, when it reached 24.8 L/year/person. These increases are assumed to be the result of desire for a healthier lifestyle and concerns about radioactive materials caused by the Fukushima Daiichi nuclear power plant accident in 2011^3^.

Water-quality standards for drinking water are regulated by the World Health Organization (WHO)^4^, and standards are also set in the US^5^, the European Union (EU)^6^, Japan^7^, and other countries. In Japan, tap water-quality standards, including metals, pesticides, organic pollutants, as well as microbial water-quality parameters, are set under the Water Supply Law. Water quality is continuously monitored, and its quality rarely exceeds standards^8,9^. Japanese tap water is supplied directly to consumers from a single source/water purification plant, or multi-sources, e.g., blending surface water and groundwater. The source and amount of supply would be modified due to demand changes and sudden natural/industrial disasters. Moreover, the fact that one municipality management implies multiple tap water-supply companies complicated supply systems. Under such circumstances, unpredictable factors may contribute to the quality of tap water in Japan. Moreover, once the water leaves the water purification plant, water quality may deteriorate due to the influence of piping and material loss. However, it has yet to be investigated whether the quality of the tap water supplied would be worse than raw or purified water. Furthermore, since tap water is used continuously in the same environments, such as homes, offices, and factories/plants, it does not appear that users are concerned about and compare the quality of water purification plants and tap water in residence areas or locations of their own. In contrast, although the durability of water pipes in Japan is specified as 40 years, actual usage ranges from 40 to 70 years^10,11^. We assumed that the consumers are concerned about the water quality effects of aging pipes.

In Japan, the water supply coverage ratio is over 98%, supplying safe tap water for drinking^12,13^. However, we are facing the following water issues:

*Water supply administration, replacement of water pipes:* Many of the water pipes are now out of durable time and need to be replaced. However, most of the local governments that are entrusted with water supply administration are financially straining, so it is not easy to replace water pipes or even find out records of when water pipes were installed. In addition, with finances are severely due to COVID-19, there are situations that water charges are not being collected, making it even more difficult to maintenance and management^14–17^.

*Natural disasters:* In recent years, natural disasters such as torrential rainfall and earthquakes have been occurring frequently. There are concerns about the deterioration of water quality due to damage to aging water pipes.

*The privatization of water supply services:* In Japan, privatization of water services has been legislated in 2018. There are concerns that privatization will lead to worsening of water quality management and non-disclosure of water quality information.

The above problems can also cause future changes in water quality from both natural and human activity aspects. Thus, it is important to measure the current conditions and make them available to the public.

Drinking-water hardness is an important issue involving both suppliers and consumers^18^. Hardness is very much linked to taste, and it is likely that consumers sense changes in water hardness^4,19^. Furthermore, when it comes to living in a different area, water hardness is important and of significant interest globally on the occasion of soap-foaming, washing machine, dishwasher maintenance and access to low-mineral water for children. In addition, the preferred water hardness varies depending on the cuisine^20^. For example, hard water is preferred for Western cuisine that take soup stock from meat, while soft water is suitable for Japanese cuisine that kombu (kelp) and bonito are used.

Hardness itself is one of the most frequently discussed issues of water quality. Hardness results mainly from the presence of dissolved calcium and magnesium ions, which are unregulated elements^4,21^. In water quality analysis, unregulated as well as regulated impurities are measured. Poisonous material contents are universally monitored and the data are being disclosed. However, non-hazardous materials that are ubiquitous in the environment (i.e., elements that do not attract attention), such as hardness and major cations/anions, tend to be considered and published as a single flat value for wide regions, leading to lack of research on their detailed distribution.

Japan is generally classified as a soft-water country, though no large-scale survey exists to support this classification. Although comprehensive research analyzed tap water in the EU region^22–24^, no reports exist on tap water quality in other countries including Japan. Additionally, the quality of the water supply in Japan has been analyzed separately by local governments and suppliers, and the results were independently released to the public. Accordingly, no option exists but to rely on information that relates only to specific areas. The problem is that these results were analyzed in different laboratories with varying measurement accuracy. It is thus difficult to compare and gain an overall impression of water-quality to make parallel comparison. No data were ever published that provides unified results at a nationwide scale. To evaluate water quality, information is needed that centrally indicates data analyses using the same method.

In this study, we conducted a comprehensive analysis of Japan using a uniform methodology with samples collected and analyzed within a certain time frame to assess the hardness of Japanese tap water in terms of its regional characteristics, and considered its relationship to water treatment plants, and the influence of piping. We collected tap water samples from 665 points throughout Japan and analyzed them at a single laboratory to investigate the distribution of tap water hardness in Japan. Then, using a unified index with fixed survey points, we evaluated hardness fluctuations in the distribution system from raw to tap water. Moreover, to characterize Japanese tap water, we also collected tap water that was available in various countries and reported basic data on the distribution of tap water hardness and major cations, such as calcium (Ca), magnesium (Mg), potassium (K), and sodium (Na).

## Results and discussion

### Distribution of tap water hardness worldwide

**Figure 1:**
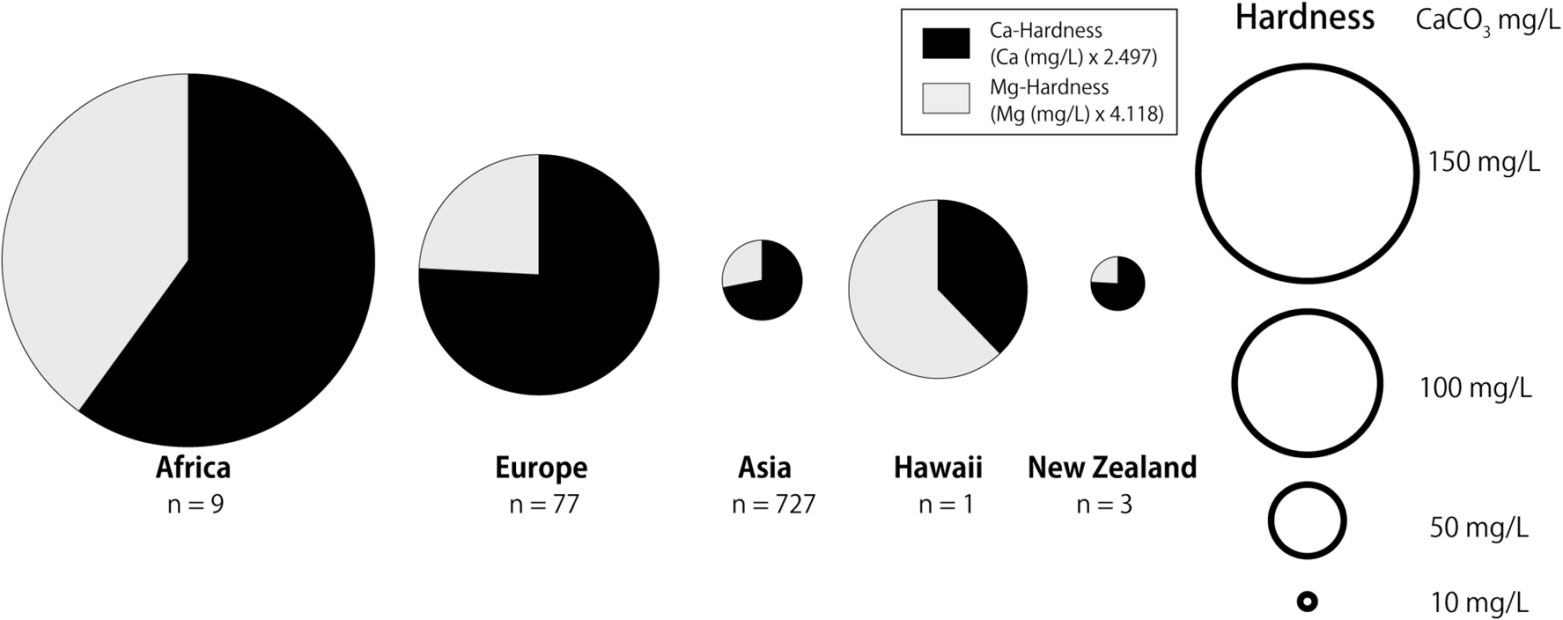
Graphic distribution of tap water hardness and the proportion of Ca and Mg-hardness by study area. The area of circles is proportional to hardness. *n* the number of samples.

From 2017 to 2020, tap water samples were collected from 28 countries in Africa, Asia, Europe, Hawaii, and New Zealand. We were unable to collect tap water samples from the mainland of the Americas during the study period. The results of tap water hardness by study area are summarized in Fig. 1 and Table S1 in the Supplementary Information. The mean hardness in Africa and Europe was above 100 mg/L. Except for Hawaii, the contribution of Ca-hardness to total-hardness was greater than that of Mg.

**Figure 2:**
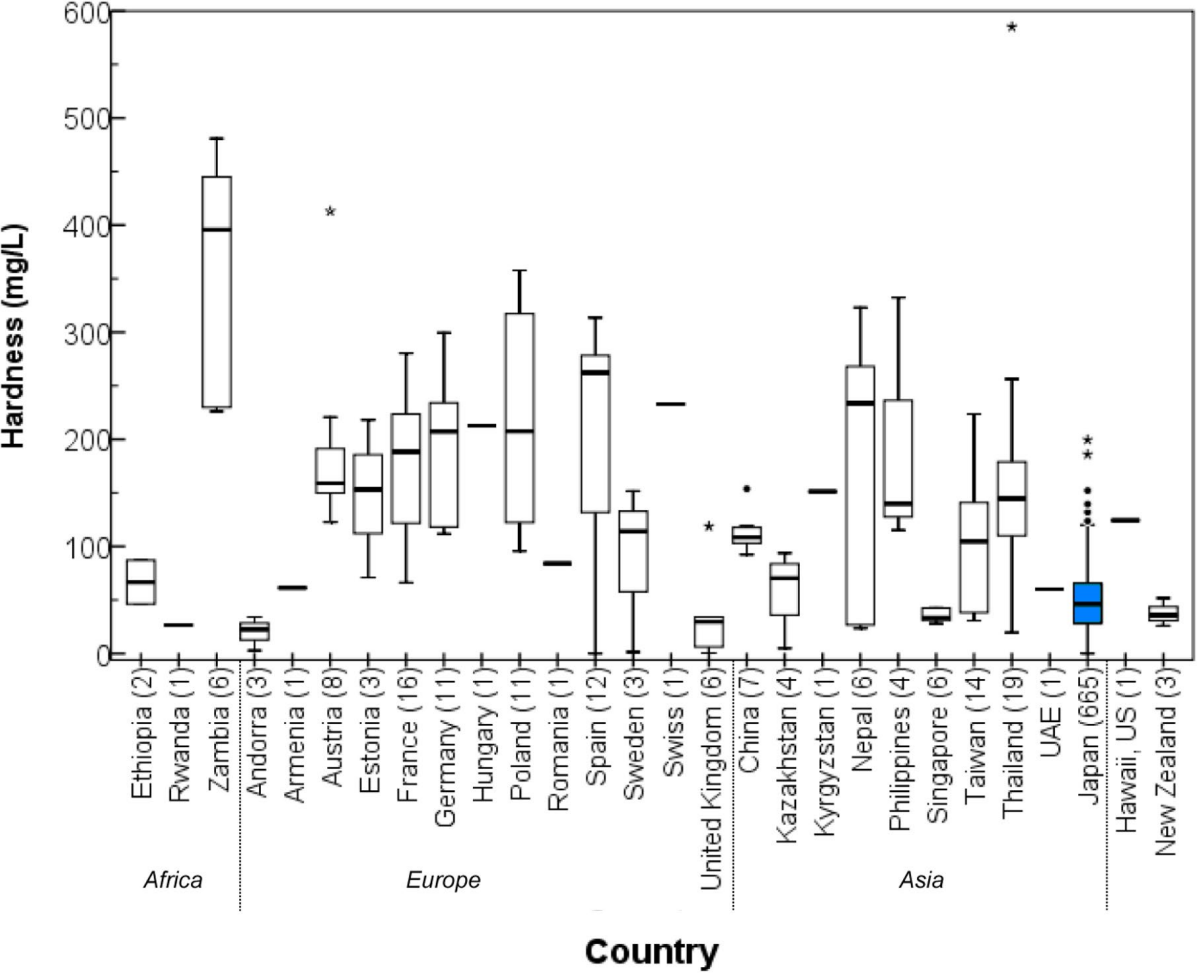
Boxplot showing the distribution of tap water hardness by country. The box shows the interquartile range, with a thick line at the median. The whiskers represent the non-outlying extraquartile range. Outliers are shown as circles (near outliers) or asterisks (far outliers). The number of samples for each country is indicated in parentheses.

Figure 2 shows a boxplot of water hardness for the collected tap water by country. The concentrations of hardness and major cations are given in Table S2. For example, the mean hardness values were 178 ± 68 mg/L (1σ SD, n = 16) in France, 187 ± 71 mg/L (n = 11) in Germany, 362 ± 112 mg/L (n = 6) in Zambia, 125 mg/L (n = 1) in Hawaii, 164 ± 122 mg/L (n = 19) in Thailand, 114 ± 20 mg/L (n = 7) in China, 37.8 ± 13.1 mg/L (n = 3) in New Zealand, and 48.9 ± 25.8 mg/L (n = 665) in Japan. For the United States, the data were collected only in Hawaii. Hawaiian tap water hardness was 125 mg/L, while the mean values in New York City and San Francisco for 2019 were reported as 29 and 47 mg/L, respectively^25,26^. These values are not representative of the US as a whole because the mainland and the Hawaiian islands exhibit different hardness levels. In Toronto, Canada, drinking-water hardness was reported as 128 mg/L in 2018^27^. It is considered that the water hardness in the continental Americas also will be widely distributed. In some areas of high hardness, water may be treated during the water purification process to reduce hardness, or water purification and hardness reduction equipment may be installed in the water-supply system of homes^28^. According to a classification provided by the WHO, water hardness below 60 mg/L-CaCO_3_ is generally considered to be soft, 60 to 120 mg/L is considered to be moderately hard, 120 to 180 mg/L is hard, and above 180 mg/L is considered to be very hard^29^. As such, the water hardness in Japan was ranked as soft.

Guideline values for water hardness have not been set by the WHO guidelines^4^, drinking water guidelines by the US Environmental Protection Agency^5^, EU Drinking Water Directive (98/83/EC)^6^, and 57 countries and territories set a value^30^. In our study, the following countries have set regulatory or guideline values: Ethiopia (300 mg/L), Rwanda (treated potable water: 300 mg/L, natural potable water: 600 mg/L), Zambia (500 mg/L), China (450 mg/L), Japan (300 mg/L), Kazakhstan (7.0 meq/L, equivalent to 700 mg/L), Kyrgystan (7.0, mmol/L equivalent to 700 mg/L), Nepal (500 mg/L), Philippines (300 mg/L), Taiwan (300 mg/L), Thailand (300 mg/L), UAE (300 mg/L, depends on territory), Armenia (7.0 mmol/L, equivalent to 700 mg/L), New Zealand (200 mg/L, 100–300 mg/L as taste threshold). None of the countries exceeded the guideline values in the country averages (Table S2).

Some of the tap water samples we collected from Thailand and Nepal exhibited a yellowish-brown color. Furthermore, selected samples from China and Taiwan developed moss during storage in the laboratory. We do not know if these samples were drinkable, but this development indicated that the sanitary conditions of tap water varied globally.

Tap water is derived from various sources, e.g., surface (river) water, groundwater, spring water, lake water, and artificial reservoirs. Japanese tap water can be characterized as follows: 48% dam water, 25% river water, 1.4% lake water, 3.5% subsoil water, and 20% groundwater^9^. Thus, tap water sources in Japan include a higher proportion of surface than groundwater. Contrastingly, most European nations’ tap water is derived from groundwater^24^. Alkalinity levels based on major cations such as Ca, Mg, and Na are higher among groundwater sources compared with surface water sources in general, resulting in higher hardness in the case of the groundwater sources^24,31^. Moreover, tap water supplied from limestone geologies (e.g., Europe) exhibits a high Ca content and, as a result, becomes hard water. In Japan, one-third of the total surface soil comprises igneous rock, and water eluted from igneous basement areas is characterized by low Ca concentrations. The mountainous regions of Japan are primarily comprised of sedimentary rock, sandstone, chert, and limestone^32^. Conversely, the lowland plains of the country is comprised of alluvial fans, flood plains, and deltas, with Pleistocene terraces at slightly higher altitudes. The plains include significant levels of river sediment, resulting in unstable and soft ground. The average precipitation in Japan is high, around 1600–1800 mm, whereas the world average is about 1000 mm^33^. Since about 70% the country consists basically mountains and its topography is steep, rainfall run-off rapidly^32,34,35^. Japanese rivers are shorter (maximum length: 370 km) and steeper (average slope: 0.44%) than their continental counterparts, and they flow rapidly down the mountains, across the plains, and into the ocean^32,35,36^. For these reasons, the residence time of river water tends to be shorter, rendering water hardness lower than in other countries. According to this comprehensive survey, we could clearly and quantitatively show the distribution of tap-water hardness.

### Tap water hardness in Japan

The water hardness of Japanese tap water in all the collected samples (n = 665) ranged from 0.101 (Naha Airport, Okinawa) to 200 mg/L (Kamisato highway rest area, Saitama). The mean value was 48.9 ± 25.8 mg/L, and the median was 46.0 mg/L. Water hardness, as well as additional information, are summarized in Table S3. A histogram of hardness for all the samples (n = 665) is shown in Fig. S1. According to our data, the bulk of the analyzed samples were characterized as soft, i.e., 65 samples with a hardness of 0 to 20, 204 for 20 to 40, 199 for 40 to 60, 117 for 60 to 80, 62 for 80 to 100, 12 for 100 to 120, 3 for 120 to 140, 1 for 140 to 160, and 2 for 180 to 200 mg/L (Fig. S1). Thus, Japanese tap water was classified as soft water that was widely distributed throughout the country. The pH of all samples that could be measured was in the range of 6.0 to 7.0.

**Figure 3:**
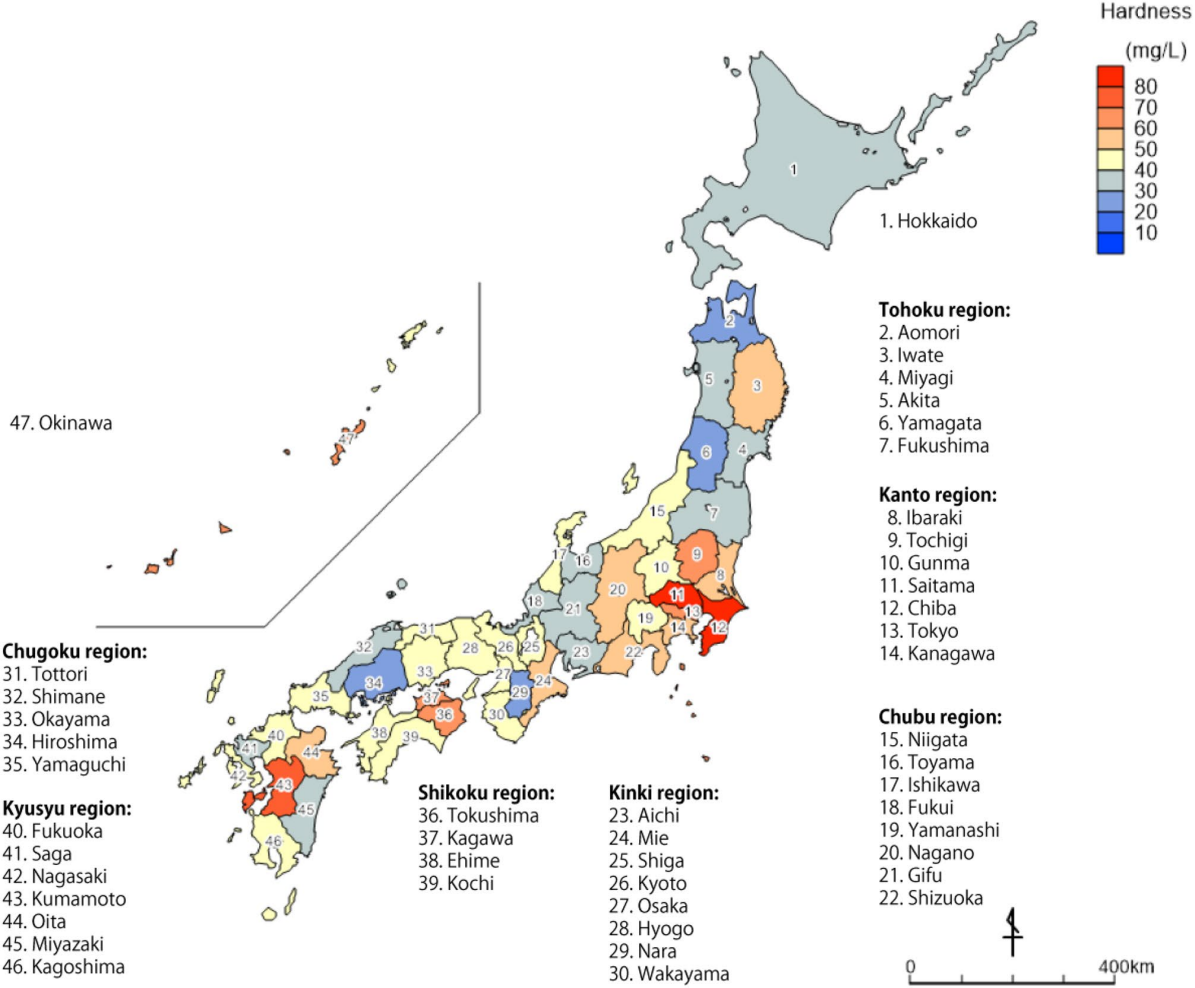
Map of Japan showing the distribution of water hardness (prefecture average). No. 1: Hokkaido, 2–7; Tohoku region, 8–14; Kanto region, 15–22; Chubu region, 23–30; Kinki region, 31–35; Chugoku region, 36–39; Shikoku region, 40–46; Kyusyu region, 47; Okinawa. The map was self-drawn by using Adobe Illustrator CS6.

**Figure 4:**
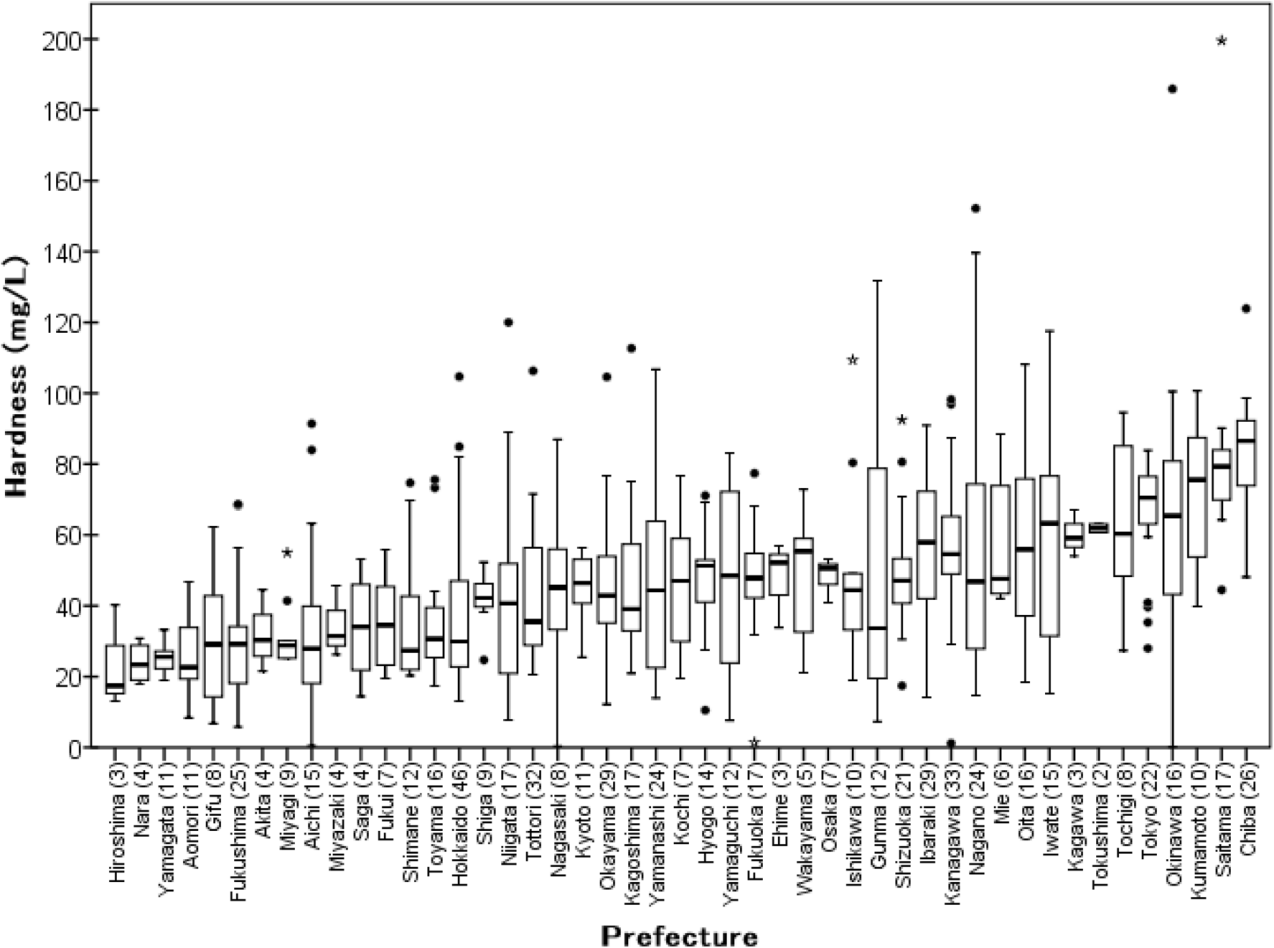
Distribution of water hardness in tap water based on increasing prefecture averages. The box shows the interquartile range, with a thick line at the median. The whiskers represent the non-outlying extraquartile range. Outliers are shown as circles (near outliers) or asterisks (far outliers). The number of samples for each prefecture is indicated in parentheses.

Among 661 of all 665 samples (excluding tap water taken from trains, n = 4), the distribution of mean hardness in all 47 prefectures in Japan is shown in Fig. 3, and the boxplot is shown in Fig. 4. The hardness and concentrations of Ca, K, Mg, and Na for each prefecture are summarized in Table S3. Chiba Prefecture exhibited the highest hardness with 83.4 mg/L, followed by Saitama with 82.8, Kumamoto with 72.2, Okinawa with 68.1, and Tokyo with 65.8 mg/L (Figs. 3, 4, Table S3). The lowest was 23.5 mg/L in Hiroshima Prefecture. Water hardness in the Hokkaido and Tohoku regions, which are located on the northern side of Japan, was lower and tended to be high in the Kanto region (Fig. 3). Since Hokkaido and Tohoku are snowfall regions, the raw water contains snowmelt water, and, as such, the hardness of water is considered as being low. Chiba, Saitama, and Tokyo, which are particularly high in the Kanto region (Ibaraki, Tochigi, Gunma, Saitama, Chiba, Tokyo, and Kanagawa), are in the center of the Kanto region, where the Tone River serves as the main water source for these cities. The Tone River basin area is the largest in Japan (16,840 km^2^) and serves approximately 12 million people (approx. 1/10 of the Japanese population)^37^. This river flows through an area where human activities thrive and, accordingly, is an important water source for human activities in the Kanto region. As such, this area includes many sources of water pollution from human activities^38^. Moreover, these three prefectures (Chiba, Saitama, and Tokyo) are located downstream of the Tone River; the linear distance from the headwater to sluice gates is more than 150 km and exhibits a long residence time before being taken in as raw water. The concentration of major ions such as Ca in the river increases in downstream areas due to human activities and urbanization^39–41^. In addition, the major aquifers of the Kanto plain comprise quaternary sediments and, in a geological context, represents a new environment in which Ca and Mg are easily eluted^35,42^. For these reasons, the tap water hardness in the Kanto region is considered to be relatively hard.

**Figure 5:**
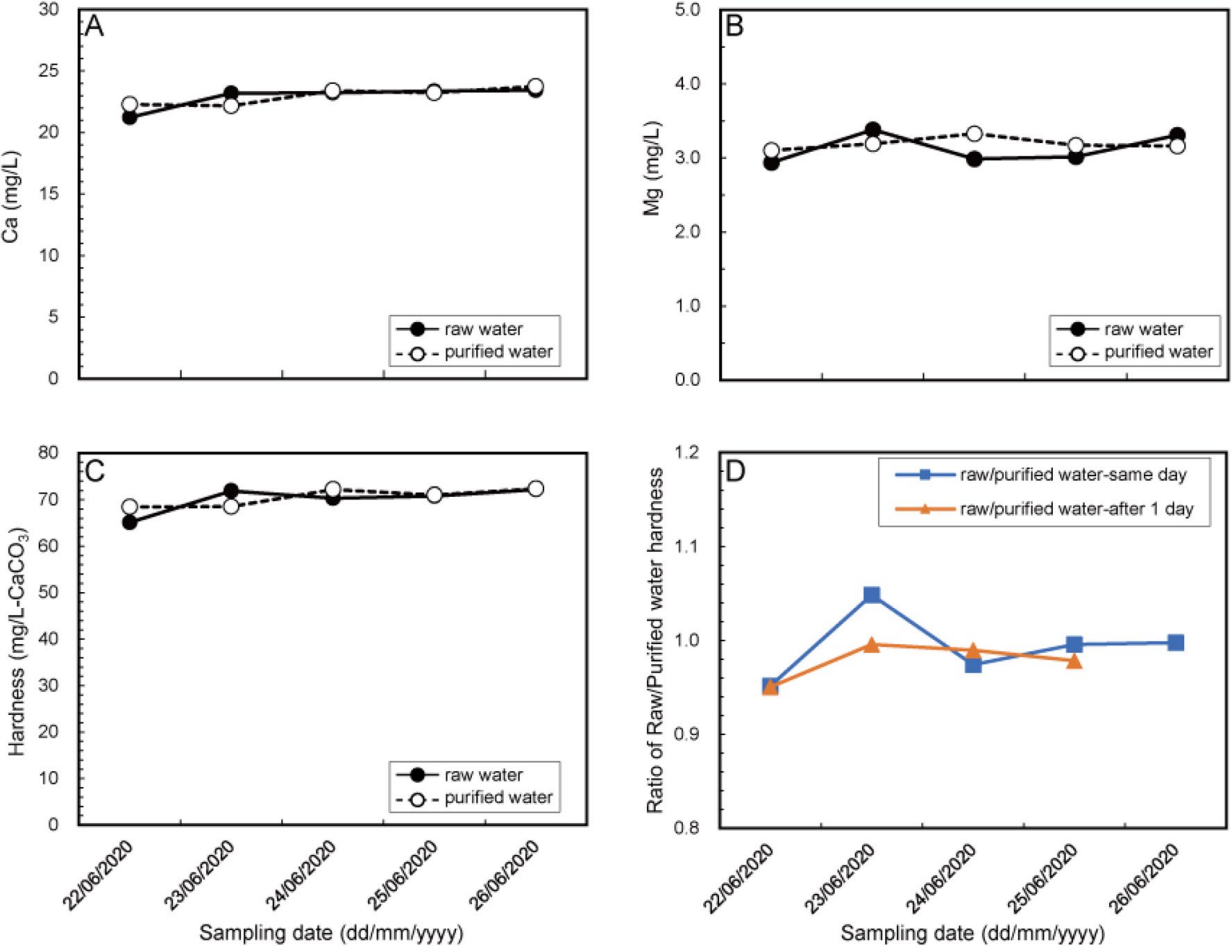
Daily changes in (**A**) Ca, (**B**) Mg, and (**C**) water hardness concentrations in raw and purified water at the Asaka Water Purification Plant. (**D**) Ratio of raw/purified water hardness (R/P ratio). Blue line and squares indicate the R/P ratio, raw and purified water collected on the same day. The orange line and triangles indicate the ratio of raw water, and purified water 1 day later considering the purification time.

Kumamoto exhibited a generally high concentration of major cations such as Ca and Mg in tap water samples (Table S3). Kumamoto is the only prefecture in Japan with a higher proportion of groundwater in its tap water (79%) than surface water^43^. Kumamoto groundwater passes through the permeable pyroclastic flow deposits of Mount Aso (Aso-1, 2, 3, 4) and springs up rich in ionic species^42,44^. Due to the characteristic geological environment, tap water exhibits a high hardness in Kumamoto.

Okinawa’s tap water hardness concentrations ranged from 0.101 to 186 mg/L; the mean and median were 68.1 and 65.4 mg/L (n = 16), respectively. The central and southern areas in Okinawa exhibit Ryukyu limestone geology. River and groundwater sources flow through this limestone, rendering rivers and groundwater abundant in Ca^45^. Thus, the water in this area exhibits high hardness. In the city of Naha, Okinawa, tap water is distributed from two water purification plants, one of which uses surface water as a water source, while the other uses groundwater as a primary water source^46^. Hardness concentration varies based on which of the two water sources is distributed evenly within the same city; therefore, the distribution of water hardness can increase accordingly. Moreover, on the Amami Islands (including Okinawa), which are comprised of Ryukyu limestone, Ca-removal equipment may be installed in water purification plants to prevent Ca from entering distribution pipes, where it can cause scaling and clogging^42,45–48^. Additionally, seawater desalination systems were installed in selected purification plants, rendering water hardness lower than the average level. Although the mean hardness of Okinawa Prefecture as a whole was higher, the minimum value of 0.101 in our study was observed at Naha Airport. This suggested that the airport exhibited its own water purification system. In the case of Okinawa, selected water treatment systems were installed due to the area’s characteristic geology and its Ca-rich water, indicating the wide distribution of hardness.

The distribution of hardness was broad within the same prefecture, i.e., 48.1 to 124 mg/L in Chiba (n = 26) (where hardness was the highest in this study), 39.8 to 101 mg/L in Kumamoto (n = 10), and 0.101 to 186 mg/L in Okinawa (n = 16). The Kamisato highway rest area, where the maximum hardness value (200 mg/L) was observed, is located in Saitama Prefecture, which exhibited the second-highest hardness (82.8 mg/L). Thus, water hardness varied from one area to another in the same prefecture. Tap water hardness in a private home in Chiba was 85.8 mg/L, whereas well water was 236 mg/L for the same house. The hardness of well water was higher than that of tap water because the former was supplied primarily by groundwater. If the hardness value is higher than the mean value, well water (groundwater) may be used or mixed as a water source through the supply process. In addition, hardness may vary significantly even within the same region. Of the four samples from Nagoya city (Aichi Prefecture), water hardness was 0.515 mg/L at one hotel, 20.0 mg/L at a different hotel, 12.7 mg/L at a third hotel, and 26.8 mg/L at Nagoya station, respectively. The mean hardness in Aichi was 33.7 mg/L. In the case of the hotels, it is assumed that they might have installed their own water purification systems for hospitality purposes (as noted in the case of Naha Airport). Thus, the observation exists that variations in water hardness, and thus mineral content, occurred within the same city. Moreover, geology differences also appear to influence water quality and may be a factor in increased deviations. It is considered that the observed regional differences reflected factors such as the supply of tap water from various water sources, as well as the blending of water from different sources and in the supply process.

Japan is now in time of replacing water pipes and the replacement is being done sequentially. Continued use of deteriorated water pipes may change water quality because of its breakage and corrosion. Furthermore, in areas where surface water is the main source of tap water, especially in downstream areas, prospective urbanization and dense population may cause changes in water quality, resulting in higher hardness. Thus, continuous monitoring is required to sustain current quality. This tap water dataset is considered a valuable resource for understanding water quality in Japan as of 2020. Due to the low number of samples and non-uniform coverage of sampling points, however, obvious limitations exist in the interpretation of the data collected for this study. Moreover, given the wide range of maximum and minimum water hardness values, the reader should take care not to assume that the characteristics of these samples are representative of a prefecture and/or region as a whole. A more micro-point-based and expansive study is necessary for gaining comprehensive results. Such an approach can be adopted in future studies.

### Changes in water hardness from raw to tap water

The mineral content of tap water can vary significantly based on the season and even daily^49^. However, no data were reported observing how much hardness can change. To clarify changes in water hardness in detail, fluctuations in hardness were evaluated for water as raw water, water in a purification plant, and a tap water supply. We suggest in the original index the ratio of raw to purified water hardness (R/P) and that of tap to purified water hardness (T/P) as a means for evaluating the dynamics of water hardness, based on the hardness of raw water, purified water, and tap water. When the ratios of R/P and T/P were 1.0, it is judged that no change in water hardness occurred. Subsequent differences from 1.0 were used to evaluate an increase or decrease in hardness.

First, we evaluated the daily water hardness changes in raw and purified water at the same point. We collected raw and purified water at the Asaka Water Purification Plant, which demonstrates the largest water purification capacity and supply capacity among Tokyo’s water treatment facilities. These water samples were collected for 5 days from June 22 to June 26, 2020; no dramatic changes occurred in weather conditions during this period. The daily dynamics of Ca, Mg, and hardness concentrations are shown in Fig. 5. Over 5 days, the hardness of raw and purified water was stable; coefficient of variation (CV) was 3.6% and 2.4%, respectively (Fig. 5C). The R/P ratio was almost 1.0 over 5 days (CV was 3.2%), indicating no change in hardness during the water purification process (Fig. 5D, blue squares and line). The Asaka Water Plant purifies water 24 h a day. The R/P ratio of purified water after 1 day, which was shifted by 1 day, considering the water purification time and the residence time at the purifying plant (e.g., the ratio of raw water hardness on June 22 to purified water hardness on June 23), was almost 1.0 (CV 1.8%) (Fig. 5D, orange line). Based on these results, the hardness of purified water was derived from the hardness of raw water. The fluctuation in Ca and Mg concentrations were also low; CV was 3.6% for Ca and 5.8% for Mg in raw water and 2.7% for Ca and 2.3% for Mg in purified water, respectively (Fig. 5A,B). This indicated that the observed small changes in raw water hardness were largely dependent on the dynamics of Mg concentration.

**Figure 6:**
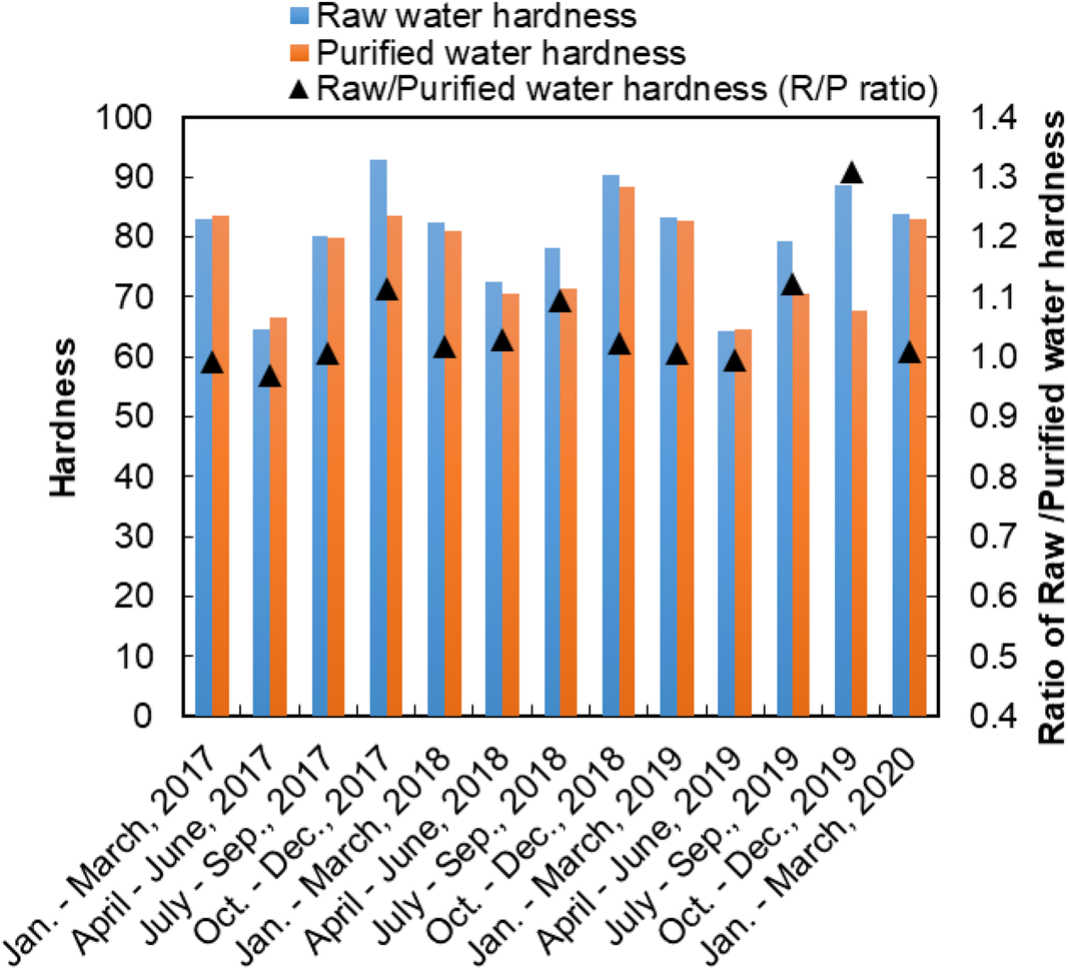
Seasonal changes in hardness and the R/P ratio at the Asaka Water Purification Plant. The ratio of raw/purified water hardness (R/P ratio) were calculated from municipal reports^50–53^.

Next, we evaluated seasonal changes based on water-quality data published by the Asaka Water Purification Plant and provided by the Tokyo Metropolitan Government^50–53^. Figure 6 indicates seasonal changes in both raw and purified water hardness from the Asaka Water Purification Plant, as well as the R/P ratio calculated as an indicator of hardness that changes within the purification system, from raw water at the sluice gate to purified water. The R/P ratio was almost 1.0 and exhibited an 8.3% CV during the course of the 3 years. The variation range was 1.0 to 1.1, except for data from October to December 2019, meaning the hardness may have changed by ± 10% in the process of water purification. However, almost no difference was found between raw and purified water. Thus, the fluctuation of hardness in the purified water depended on raw water hardness. In the spring (April to June), the water hardness tended to decrease. A similar trend was observed for other tap water taken from a water purification plant in the Tone River system^54,55^, suggesting it had been caused by snowmelt in the mountains upstream of the Tone River.

**Figure 7:**
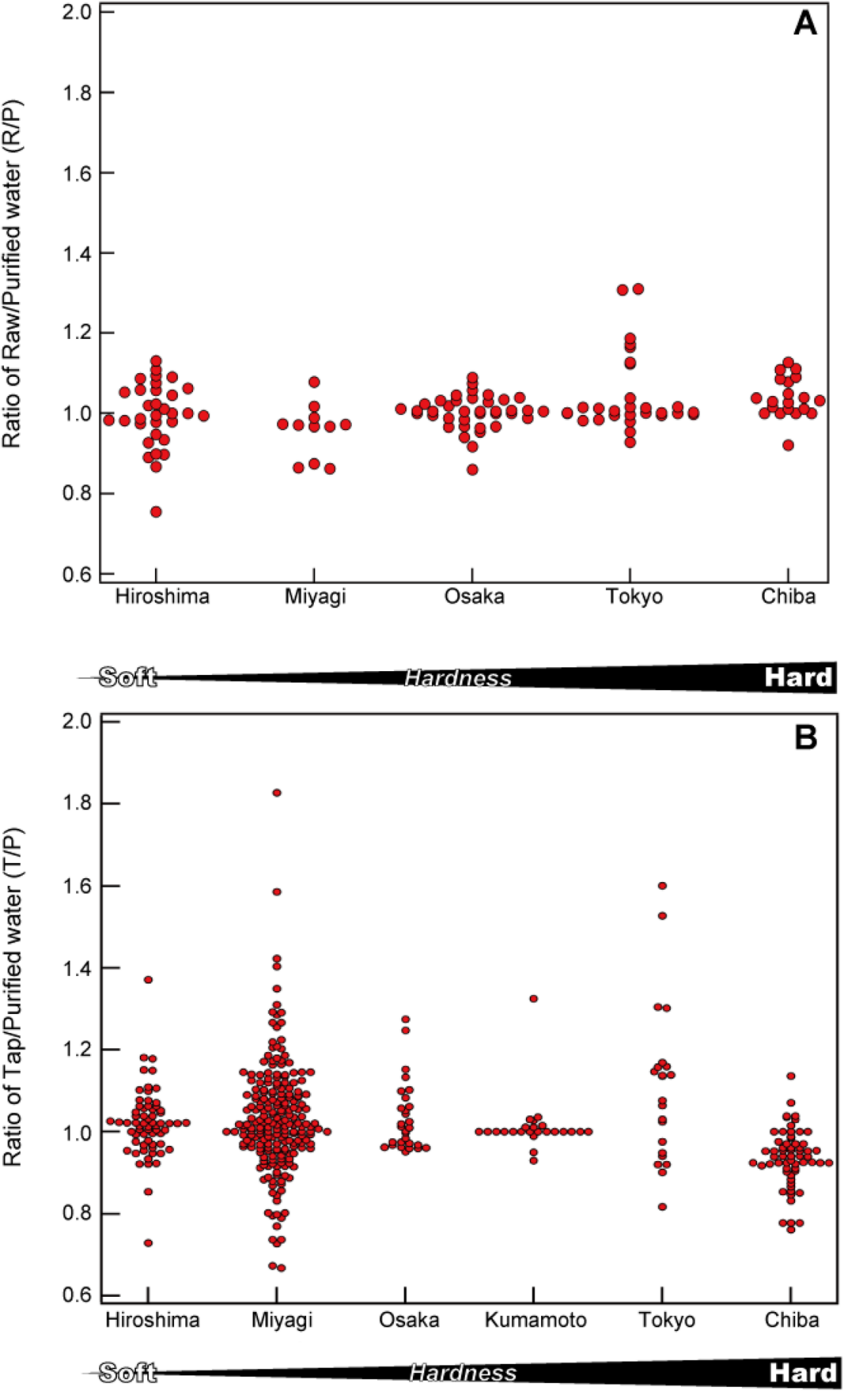
Distribution of R/P and T/P ratios in Hiroshima and Miyagi (relatively soft water), in Osaka (water with average hardness), and in Tokyo and Chiba (relatively hard water). For Kumamoto prefecture, only purified water and tap water data were available. (**A**) Represents R/P ratio; (**B**) represents T/P ratio. These data were calculated from municipal reports^54–60^ and our data.

To evaluate the dynamics of water hardness in Japan, we compared the changes in hardness between raw, purified, and tap water. We selected Hiroshima and Miyagi as exhibiting soft water, Osaka as exhibiting an average water hardness, and Tokyo and Chiba as exhibiting high water hardness (Fig. 7). Also, to estimate changes in the distribution process, the ratio of tap/purified water hardness (T/P) was evaluated (Fig. 7B). Data were obtained from water-quality reports published by local governments in 2018 and 2019^52,53,55–60^. These data indicated a direct one-to-one correspondence between raw to purified water and purified to tap water were used for the evaluation. The data were limited, however, because obtaining a series of data ranging from raw to tap water in the same location was difficult.

In a case where the water quality of the source river exceeds the standard pH, lime can be added to adjust pH during water treatment. In such a case, the water quality of raw and purified water will not match, and the difference between the R/P ratio = 1.0 becomes large. However, assessing whether or not pH adjustment was carried out is impossible because this is not disclosed to the public.

The variation in R/P ratio ranged from 0.8 to 1.2, indicating a ± 20% change in hardness during the water purification process, regardless of the water hardness level (Fig. 7A). At points in Tokyo where the R/P ratio was as high as 1.2 to 1.4, different values compared with other sites resulted from the installation of a different water purification method using a membrane filtration system. The main reason for this slight difference in hardness between raw and purified water was considered to have been the water treatment time. For example, the water treatment time at a Tokyo water purification plant is approximately 8 h. If water is sampled during a period when the water quality fluctuates significantly (e.g., during heavy rainfall or a typhoon), the hardness between raw and purified water may differ. Regardless, although the hardness fluctuated approximately ± 20% during the water purification process, it generally remained stable.

Figure 7B shows the T/P ratio, an indicator of hardness that changes within the distribution system, from water in purification plants to tap water supply. The variation range was primarily 0.8 to 1.2, indicating a ± 20% change from purification plant to tap water. It should be noted that since the hardness value of Hiroshima and Miyagi was low, the T/P ratio increased with only a slight difference in hardness between purified and tap water.

Two possible factors can change the hardness between purified and tap water: (1) the impact of the supplying distribution pipe, and (2) the mixing of tap water with water of a different quality. The bulk of water-supply pipes in Japan are ductile cast iron pipes in which the main components are iron and carbon. It is unlikely that these components will directly affect water hardness. While Ca may cause scale deposition in pipes in hard-water areas above 200 mg/L^4^, the formation and dissolution of scale is unlikely, considering Japanese tap water hardness (mean value 49 mg/L-CaCO_3_). As such, water-supply pipes are not considered to affect the water hardness in Japan. However, the distribution system in Japan used by purification plants for delivering tap water is complicated. Once in the distribution system, the distribution pipes branch and repeatedly merge, thus mixing multiple water-types within the distribution system in the process of providing tap water. In addition, the water supply from purification plants is not constant over 24 h, and water with different hardness levels are mixed during the water supply process. Since multiple water sources are blended due to the branching and merging of distribution pipes, changes in hardness during distribution can occur. For these reasons, hardness can slightly change between purified and tap water. Water hardness also reflects the geological properties of the area with which the water has been in contact^18^. Accordingly, the hardness of supplied tap water is essentially derived from purified water hardness, i.e., from raw water hardness.

The hardness variation range can change ± 20% during water purification processes and in the tap water-supply systems in any hardness region of Japan. It was found that a low possibility of contamination from the pipes and the tap water hardness derived from the quality of the raw water. Since surface water is the main source of tap water in Japan, raw water quality tends to be exhibited in tap water. If tap water shows a particularly higher hardness than usual, the assumption exists that the quality of the raw water somehow differs from the usual environment. The hardness of tap water in Japan is considered to be stable, with little or no daily change, although seasonal variations can be observed throughout the year.

## Conclusion

We comprehensively investigated the hardness of tap water in Japan on a nationwide scale from 2017 to 2020 and collected tap water from 817 points including 27 other countries. Compared with 27 countries, tap water in Japan exhibited low-mineral content and presented as soft water. Japanese tap water hardness was 48.9 ± 25.8 mg/L (mean ± 1σ, n = 665) and that was classified as soft water, but its distribution was large throughout the country. The change of tap water hardness in the Japanese supply system and revealed that the hardness variation range can change ± 20% during water purification processes and in the tap water-supply systems. A comparison of the variations in hardness showed that there was no daily fluctuation in hardness and it was generally stable. In Japan, the hardness of tap water was not affected by piping but by the quality of the raw water.

The results represented here is considered a valuable resource for not only researchers but also stakeholders and end-consumers because it represents the comprehensive Japanese-wide survey of tap water quality with a uniform sampling period and contains the largest number of sites in the currently available dataset.

## Methods

### Sample collection and analysis

**Figure 8:**
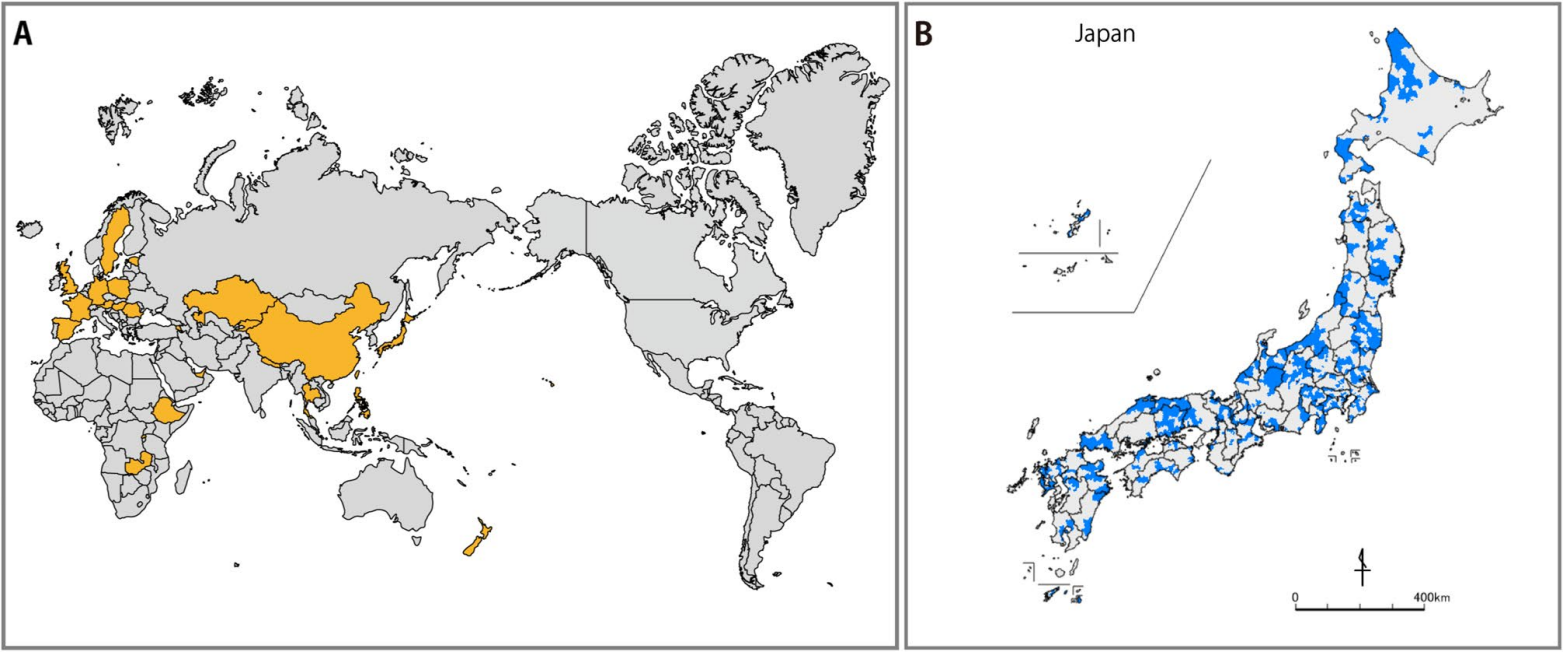
Locations of the tap water samples: (**A**) a map of the study countries (n = 28); (**B**) the location of sampling cities in Japan (n = 665). For the United States, the data were collected only in Hawaii. The figure was self-drawn by using Adobe Illustrator CS6.

A total of 665 tap water samples from all 47 prefectures of Japan were collected from 2017 to 2020. The sampling strategy was directed at end-consumers. As such, we collected samples from private homes and public spaces (offices, restaurants, public facilities, train stations, airports, or highway service stations) that are supplied via a public distribution system throughout Japan without distinguishing the private/public facilities. The collection of tap water was preferentially sampled from representative and populous cities, as well as from a large number of local municipalities to ensure that sampling sites did not overlap. When collecting tap water, details about the condition sources were not specified; this was only performed for samples taken from water pipes. As such, whether the water samples were single or multiple-source samples was not specified. To include many different areas, sampling was conducted not only by our research team but also by other researchers, colleagues, and friends who supported the importance of this study. The collection areas are shown in Fig. 8. Furthermore, to characterize Japanese tap water, this type of sample was also collected during the study period from 152 points in 27 countries (excluding Japan) with the help of colleagues. Samples were collected from ordinary taps, although no survey of whether the water was drinkable was conducted. This study focus on the geographical difference of chemical characteristics and quality in tap waters, featuring water hardness and its fluctuations during the distribution process, which all water samples were collected and analyzed under the authorization of each site. In addition, humans are not directly involved in the present study.

Tap water samples were collected directly from taps and stored in polyethylene bottles. Before collection, to avoid collecting stagnant water in the distribution system, water was run from the tap for several seconds. The bottles used were rinsed several times with the tap water; samples were stored as filled bottles to prevent air bubbles as much as possible. Whenever possible, the water pH was measured in the field using a Merck MQuant StripeScan. No filtration was performed during sampling.

Collected water samples were analyzed for concentrations of Ca, Mg, K, and Na using inductively coupled plasma optical emission spectroscopy (ICP-OES) (Agilent ICP-OES 720). The detection limit for each element was 0.005 mg/L.

To investigate the dynamics of water hardness, raw and purified water samples were collected for five consecutive days from June 22 to June 26, 2020. The river water was collected as raw water samples from the Akigase Sluice Weir (Arakawa–Tonegawa river system), one of the tap-water sources in Tokyo. Purified water from the Asaka Water Purification Plant, where the above raw water was treated, was sampled as fresh purified water from the water supply installed at the Asaka Water Purification Plant. The river water was filtrated using a 0.45 µm membrane filter. Also, we collected and referred to the raw and purified water datasets published by the water-supply utilities in each prefecture. In some cases, the sampling date and time of both water types were different; accordingly, the measurement accuracy of these datasets cannot be evaluated.

### Water hardness

Water hardness is caused by a variety of dissolved polyvalent metallic ions, predominantly Ca and Mg cations^4,29^. As such, it can be directly calculated from the analytical data, according to Eq. (1)^21,61^.

1 $${\text{Hardness }}\left( {{\text{mg}}/{\text{L as CaCO}}_{{\text{3}}} } \right){\text{ }} = {\text{ Ca }}\left( {{\text{mg}}/{\text{L}}} \right){\text{ }} \times {\text{ 2}}.{\text{497 }} + {\text{ Mg }}\left( {{\text{mg}}/{\text{L}}} \right){\text{ }} \times {\text{ 4}}.{\text{118}}.$$

The Japanese regulatory limit for tap water hardness is below 300 mg/L as CaCO_3_^7^, and this is not regulated by the WHO^4^, the US Environmental Protection Agency (USEPA)^5^, or the EU Drinking Water Directive (98/83/EC)^6^.

### Statistical analysis

The concentration of hardness varied between the different Japanese tap water samples in our study. Exploratory data analysis was used to investigate the distribution of the dataset. Data from all 665 sites in Japan and 152 sites in other countries were used in the analysis, and no points were intentionally excluded. All samples taken between 2017 and 2020 were evaluated by country or prefecture. A mean level can be skewed by extreme values in small samples. Accordingly, we report the mean, standard deviation, median, and range of concentrations. Some stats and figures are partly made by SPSS Statistics (ver. 17.0), Igor, and Excel.

## Supplementary Information

Supplementary Information.

## References

[1] Geerts R (2020). Bottle or tap? Toward an integrated approach to water type consumption. Water Res..

[2] The Mineral Water Association of Japan. *Changes in Per Capita Consumption of mineral Water* (2020). https://minekyo.net/relays/download/5/123/3/444/?file=/files/libs/444/202004021630262016.pdf (Accessed 20 November 2020).

[3] Merz S, Shozugawa K, Steinhauser G (2015). Analysis of Japanese radionuclide monitoring data of food before and after the Fukushima nuclear accident. Environ. Sci. Technol..

[4] Word Health Organization (2017). Guidelines for Drinking-water Quality.

[5] United States Environmental Protection Agency. *National Primary Drinking Water Regulations* (1996). https://www.epa.gov/ground-water-and-drinking-water/national-primary-drinking-water-regulations (Accessed 1 June 2021).

[6] The Council of the European Union. COUNCIL DIRECTIVE 98/83/EC of 3 November 1998, on the quality of water intended for human consumption. *Official Journal of the European Communities* (1998).

[7] Ministry of Health, Labour and Welfare, Japan. *Drinking Water Quality Standards* (2004). https://www.mhlw.go.jp/english/policy/health/water_supply/4.html (Accessed 20 November 2020).

[8] Japan Water Works Association. *Database of Water Quality of Aqueduct, [Database]* (2019). http://www.jwwa.or.jp/mizu/index.html (Accessed 20 November 2020).

[9] Japan Water Works Association. *Water Supply in Japan 2017* (2017). http://www.jwwa.or.jp/jigyou/kaigai_file/2017WaterSupplyInJapan.pdf (Accessed 20 November 2020).

[10] Ministry of Internal Affairs and Communications, Japan. *Legal Useful Life, Local Public Enterprise Law Enforcement Regulations, Appended Table 2 (Related to Article 14, 15)* (2001).

[11] Ministry of Health, Labour and Welfare, Japan. *Reference Material: Example of Setting Up Renewal Standard Based on Actual Years of Use* (2019). https://www.mhlw.go.jp/file/06-Seisakujouhou-10900000-Kenkoukyoku/kousinkijyun_2.pdf (Accessed 20 November 2020).

[12] United Nations Children's Fund (UNICEF) and World Health Organization. *Progress on household Drinking Water, Sanitation and Hygiene 2000–2017. Special Focus on Inequalities* (New York, 2019).

[13] Centers for Disease Control and Prevention (CDC). *Travelers' Health Tool*. https://wwwnc.cdc.gov/travel/destinations/traveler/none/japan?s_cid=ncezid-dgmq-travel-single-001 (Accessed 31 May 2021)

[14] Nippon.com. *Japan's Water Utilities Threatened by a Declining Population* (2020). https://www.nippon.com/en/in-depth/d00434/japan%E2%80%99s-water-utilities-threatened-by-a-declining-population.html (Accessed 6 June 2021)

[15] Asahi Shimbun Degital (Newspaper article). *Concerns About “Large-Scale Behavior” After a Series of Water Price Cuts in COVID-19.* 16 May, 2020, (Tokyo, 2020).

[16] The Yomiuri Shimbun online (Newspaper article). *Water Charge Reduction and Exemption 320 Local Governments. 26 August, 2020* (Tokyo, 2020).

[17] The Yomiuri Shimbun online (Newspaper article)*. 20% of Water Pipe Ledgers not Created Survey by the Ministry of Health, Labor and Welfare.* 24 May, 2021 (Tokyo, 2021).

[18] Gray NF (2008). Drinking Water Quality: Problems and Solutions.

[19] Dietrich AM, Burlingame GA (2015). Critical review and rethinking of USEPA secondary standards for maintaining organoleptic quality of drinking water. Environ. Sci. Technol..

[20] Suzuno H, Ishida H (2013). Effect of water hardness on the boiled beef, chicken and potato. J. Cookery Sci. Jpn..

[21] Weiner ER (2008). Applications of Environmental Aquatic Chemistry, A Practical Guide.

[22] Dinelli E (2012). Major and trace elements in tap water from Italy. J. Geochem. Explor..

[23] Banks D, Birke M, Flem B, Reimann C (2015). Inorganic chemical quality of European tap-water: 1. Distribution of parameters and regulatory compliance. Appl. Geochem..

[24] Flem B (2015). Inorganic chemical quality of European tap-water: 2. Geographical distribution. Appl. Geochem..

[25] NYC Environmental Protection. *New York City Drinking Water Supply and Quality Report 2019* (2019). https://www1.nyc.gov/assets/dep/downloads/pdf/water/drinking-water/drinking-water-supply-quality-report/2019-drinking-water-supply-quality-report.pdf (Accessed 20 November, 2020).

[26] San Francisco Public Utilities Commission. *City of San Francisco 2019 Annual Water Quality Report* (2019). https://sfwater.org/modules/showdocument.aspx?documentid=15360 (Accessed 20 November 2020).

[27] City of Toronto, Canada. *Drinking Water Analysis SUMMARY 2018* (2018). https://www.toronto.ca/wp-content/uploads/2019/05/8fd4-Drinking-Water-Analysis-2018-AODA.pdf (Accessed 20 November 2020).

[28] Lanz B, Provins A (2016). The demand for tap water quality: Survey evidence on water hardness and aesthetic quality. Water Resour. Econ..

[29] World Health Organization (2011). Hardness in Drinking-Water: Background Document for Development of WHO Guidelines for Drinking-Water Quality.

[30] World Health Organization (2018). A Global Overview of National Regulations and Standards for Drinking-Water Quality.

[31] Azoulay A, Garzon P, Eisenberg MJ (2001). Comparison of the mineral content of tap water and bottled waters. J. Gen. Intern. Med..

[32] Yoshimura C, Omura T, Furumai H, Tockner K (2005). Present state of rivers and streams in Japan. River Res. Appl..

[33] Ministry of Land, Infrastructure, Transport and Tourism. *Current State of Water Resources in Japan* (2019). https://www.mlit.go.jp/common/001316355.pdf (Accessed 30 November 2020).

[34] Kanto Regional Development Bureau, Ministry of Land, Infrastructure, Transport and Tourism, Japan. *Water Resources Information in the Metropolitan Area*. https://www.ktr.mlit.go.jp/river/shihon/index00000002.html (Accessed 30 November 2020).

[35] de Graaf R, Hooimeijer F (2014). Urban Water in Japan.

[36] Ministry of Land, Infrastructure, Transport and Tourism, Japan. *Land and Climate of Japan* (2007). https://www.mlit.go.jp/river/basic_info/english/land.html (Accessed 1 December 2020)

[37] Water and Disaster Management Bureau, Ministry of Land, Infrastructure, Transport and Tourism, Japan. *Tone-Gawa River System Maintenance Basic Policy* (2007). https://www.mlit.go.jp/river/basic_info/jigyo_keikaku/gaiyou/seibi/tonegawa_index.html (Accessed 30 November 2020)

[38] Ohmichi K, Miyamoto H, Ohmichi M, Machida K (2004). Inorganic elements of tap and river water in the waterase, tone and edo river system. Biomed. Res. Trace Elements.

[39] Kaushal SS (2017). Human-accelerated weathering increases salinization, major ions, and alkalinization in fresh water across land use. Appl. Geochem..

[40] Moore J, Bird DL, Dobbis SK, Woodward G (2017). Nonpoint source contributions drive elevated major ion and dissolved inorganic carbon concentrations in urban watersheds. Environ. Sci. Technol. Let..

[41] Stets EG (2020). Landscape drivers of dynamic change in water quality of U.S. rivers. Environ. Sci. Technol..

[42] Geological Survey of Japan, AIST. *Seamless Digital Geological Map of Japan 1: 200,000* (2015). https://gbank.gsj.jp/seamless/ (Accessed 7 December 2020).

[43] Kumamoto Prefecture. *Water Supply of Kumamoto, [Database]* (2019). https://www.pref.kumamoto.jp/soshiki/51/5679.html (Accessed 22 April 2020).

[44] Geological Survey of Japan, AIST. *Active Volcanoes of Japan—Aso Volcano*. https://gbank.gsj.jp/volcano/Act_Vol/aso/text/eng/exp04-1e.html (Accessed 22 April 2020).

[45] Okinawa Prefectural Enterprise Bureau. *Annual Report of Water Quality 2019 in Okinawa Prefecture* (2020). http://www.eb.pref.okinawa.jp/userfiles/files/autoupload/nenpou2019.pdf (Accessed 22 April 2020).

[46] Naha City Water Works. *Water Hardness in Naha City* (2020). https://www.city.naha.okinawa.jp/water/pax/suishitsukanri/suishitsuqa.html#cmsq7 (Accessed 20 November 2020).

[47] Okinawa Prefectural Enterprise Bureau. *Hardness Optimization of Chatan Water Purification Plant System* (2020). http://www.eb.pref.okinawa.jp/water/80/98 (Accessed 20 November 2020).

[48] Seto M (2007). Water situation in the Amami Islands of Okinoerabu, Yoron and Kikai. Amami News Lett. (Dept. Bull. Pap. Kagoshima Univ.).

[49] Postawa A (2012). Best Practice Guide on Sampling and Monitoring of Metals in Drinking Water.

[50] Bureau of Waterworks Tokyo Metropolitan Government. *Water Quality of the Asaka Purification Plant, April 2016–March 2017, [Database]* (2017). https://www.waterworks.metro.tokyo.jp/data/suigen/kekka28/j003-4.pdf (Accessed 29 June 2020).

[51] Bureau of Waterworks Tokyo Metropolitan Government. *Water Quality of the Asaka Purification Plant, April 2017–March 2018, [Database]* (2018). https://www.waterworks.metro.tokyo.jp/data/suigen/kekka29/j003.pdf (Accessed 29 June 2020).

[52] Bureau of Waterworks Tokyo Metropolitan Government. *Water Quality of the Asaka Purification Plant, April 2018–March 2019, [Database]* (2019). https://www.waterworks.metro.tokyo.jp/data/suigen/kekka30/j003-4.pdf (Accessed 29 June 2020).

[53] Bureau of Waterworks Tokyo Metropolitan Government. *Water quality of the Asaka purification plant, April 2019-March 2020, [Database]* (2020). https://www.waterworks.metro.tokyo.jp/data/suigen/kekkar01/j003-4.pdf (Accessed 20 December 2020).

[54] Bureau of Waterworks Tokyo Metropolitan Government. *Results of the Water-Quality Test, [Database]* (2020). https://www.waterworks.metro.tokyo.jp/suigen/kekka/ (Accessed 20 December 2020).

[55] Chiba Prefectural Government. *Water Quality Result, [Database]* (2020). https://www.pref.chiba.lg.jp/suidou/jousui/suishitsu/kensa/index.html (Accessed 22 April 2020).

[56] Waterworks Bureau, The City of Hiroshima. *Annual Report of Water Quality 2018 in Hiroshima City* (2019). http://www.water.city.hiroshima.jp/quality/snenpou/pdf/suishitunenpoh30.pdf (Accessed 20 November 2020).

[57] Onomichi City, Hiroshima. *Result of Water Quality 2019* (2019). https://www.city.onomichi.hiroshima.jp/soshiki/66/26585.html (Accessed 20 November, 2020).

[58] Sendai City Waterworks Bureau. *Water Quality Results for Each Purified Water and Analysis Point 2019* (2019). https://www.suidou.city.sendai.jp/nx_html/03-suishitsu/03-300-2019.html (Accessed 20 November 2020).

[59] Osaka Water Supply Authority. *Water Purification Plant Monthly Test Results, [Database]* (2020). http://www.wsa-osaka.jp/joho/siryoushu/suishitsu/suidoyosui/suidoyousuikyoukyuujigyousuishitukennsa/index.html (Accessed 19 June 2020).

[60] Kumamoto City Waterworks and SEWERAGE bureau. *Annual Report of Water Quality 2019 in Kumamoto City*. https://www.kumamoto-waterworks.jp/waterworks_article/25833/ (Accessed 19 June 2020).

[61] Baird R, Eaton AD, Rice EW (2017). Standard Methods for the Examination of Water and Wastewater.

