# Supplementary material for: A survey of monitoring tap water hardness in Japan and its distribution patterns
Source: Sci Rep. 2021 Jun 29;11:13546. doi: 10.1038/s41598-021-92949-8 (PMC8242065; doi:10.1038/s41598-021-92949-8)
Supplement: Supplementary file 1 — Supplementary Information. [file 41598_2021_92949_MOESM1_ESM.pdf]

Supplementary Information

A survey of monitoring tap water hardness in Japan and its distribution patterns

Mayumi Hori[1*], Katsumi Shozugawa[2], Kenji Sugimori[3], Yuichiro Watanabe[4,5,6]

1 Komaba Organization for Educational Excellence, The University of Tokyo, Japan
2 Department of General Systems Studies, Graduate School of Arts and Sciences, The University of Tokyo, Japan
3 Department of Biology, School of Medicine, Faculty of Medicine, Toho University, Japan
4 Department of Life Sciences, Graduate School of Arts and Sciences, The University of Tokyo, Japan
5 Integrated Human Sciences Program for Cultural Diversity, The University of Tokyo, Japan
6 Graduate Program on Environmental Sciences, The University of Tokyo, Japan

*Corresponding Author: Mayumi Hori
E-mail: cmayumi@mail.ecc.u-tokyo.ac.jp; Phone: +81-3-5454-6566

Contents:

Pages S1 to S9.
Table S1 - Tap water hardness concentration by study area
Table S2 - Results of tap water hardness and concentrations of Ca, K, Mg. and Na by country
Table S3 - Concentrations of hardness and major cations (Ca, K, Mg, Na) in all 47 prefectures in Japan.
Figure S1 - Histogram of the sampled tap water hardness in Japan (n = 665)
References

Table S1. Tap water hardness concentration by study area

Area	Hardness ($CaCO_3$ mg/L)					n
	Min	Max	Median	Mean	SD	
Africa	26.4	481	230	259	179	9
Europe	0.053	413	160	168	97.8	77
Asia	0.101	585	47.1	55.6	43.4	727
Hawaii		125				1
New Zealand	25.8	51.8	35.7	37.8	13.1	3

SD: Standard deviation (1σ), Min: minimum, Max: maximum, n: number of points

Table S2. Results of tap water hardness and concentrations of Ca, K, Mg. and Na by country.

Area	Country	n	Hardness ($CaCO_3$ mg/L)						Ca	K	Mg	Na
			Min	Max	Median	Mean	SD	regulatory/guideline value	(Mean ± SD mg/L)	(Mean ± SD mg/L)	(Mean ± SD mg/L)	(Mean ± SD mg/L)
Africa	Ethiopia	2	46.2	87.1	66.6	66.6	29	300 [1]	16.5 ± 5.5	2.96 ± 2.02	6.18 ± 3.67	7.85 ± 0.30
Africa	Rwanda	1		26.4				300*, 600** [2]	6.5	1.56	2.47	2.24
Africa	Zambia	6	226	481	396	362	112	500 [3]	86.6 ± 38.9	1.13 ± 0.31	35.1 ±5.3	19.9 ± 9.6
The Americas	United States (Hawaii)	1		125				None set [4]	19.1	3.75	18.6	27.6
Asia	China	7	92.3	154	109	114	20	450 [5]	37.0 ± 8.2	2.62 ± 0.33	5.16 ± 1.20	11.2 ± 3.0
Asia	Japan	665	0.1	200	46	48.9	25.8	300 [6]	14.2 ± 7.4	1.79 ± 1.32	3.28 ± 2.47	10.7 ± 9.7
Asia	Kazakhstan	4	4.82	93.8	70.4	59.9	38.4	700 [7]	18.6 ± 11.2	0.91 ± 0.44	4.37 ± 2.03	5.48 ± 4.38
Asia	Kyrgyzstan	1		151				700 [8-10]	48.4	1.99	7.37	11.1
Asia	Nepal	6	23.7	323	234	185	128	500 [11]	60.4 ± 42.0	11.3 ± 11.8	8.24 ± 6.21	30.6 ± 23.1
Asia	Philippines	4	115	333	140	182	101	300 [12]	39.7 ± 17.2	11.8 ± 2.8	20.2 ± 14.2	24.5 ± 11.6
Asia	Singapore	6	28.1	42.7	33.5	35.2	6.1	None set [13]	12.0 ± 2.0	3.58 ± 2.03	1.26 ± 0.37	4.47 ± 2.34
Asia	Taiwan	14	30.8	224	105	108	64	300 [14]	28.4 ± 16.5	1.59 ± 1.06	9.07 ± 6.30	13.5±14.3
Asia	Thailand	19	19.7	585	145	164	122	300 [15]	43.5 ± 24.5	7.7 ± 10.9	13.3 ± 20.1	59.5 ± 104.5
Asia	United Arab Emirates	1		60				300*** [16,17]	16.3	1.82	4.7	49.9
Europe	Andorra	3	2.76	34.2	22.6	19.8	15.9	None set [18]	7.26 ± 5.87	1.10 ±0.73	0.64 ± 0.11	10.7 ± 16.6
Europe	Armenia	1		61.2				700 [19]	14.1	2.18	6.29	13.6
Europe	Austria	8	123	413	159	192	94	None set [18]	55.3 ± 22.7	0.82 ± 1.14	13.1 ± 9.2	5.58 ± 10.3
Europe	Estonia	3	71.1	218	153	147	74	None set [18]	33.8 ± 13.1	2.49 ± 1.48	15.3 ± 14.3	6.05 ± 2.18

Europe	France	16	66.2	280	188	178	68	None set [18]	62.4 ± 25.5	1.56 ± 1.10	5.40 ± 5.29	22.7±22.7
Europe	Germany	11	112	300	207	187	71	None set [18]	56.4 ± 22.9	3.39 ± 1.46	11.2 ± 6.4	26.9 ± 9.8
Europe	Hungary	1		213				None set [18]	60.2	2.51	15.2	10.9
Europe	Poland	11	95.6	358	208	217	104	None set [18]	70.1 ± 35.6	3.49 ± 1.90	10.2 ± 4.3	17.6 ± 10.4
Europe	Romania	1		84.1				None set [18]	18.2	1.43	9.37	56
Europe	Sweden	3	1.37	152	114	89.1	78.2	None set [18]	24.5 ± 20.8	2.99 ±3.32	10.1 ±7.2	12.7 ± 12.6
Europe	Spain	12	0.053	314	262	209	111	None set [18]	62.7 ± 34.3	13.8 ± 8.4	13.9 ± 6.9	73.7 ± 41.7
Europe	Switzerland	4		232.8				None set [20]	65.6	1.4	16.8	8.7
Europe	United Kingdom	6	0.499	119	29.6	36.5	42.7	None set [18]	12.5 ± 15.5	0.85 ± 1.23	1.89 ± 0.61	8.2 ± 12.3
Oceania	New Zealand	3	25.8	51.8	35.7	37.8	13.1	200 [21]	11.5 ± 5.1	2.58 ± 1.23	2.20 ± 0.10	12.0 ± 4.8

SD: Standard deviation (1σ), Min: minimum, Max: maximum, n: number of points

* treated potable water

** natural potable water

*** Depends on territory: 300 mg/L in Abu Dhabi[16], Not set in Dubai[17]

Table S3. Concentrations of hardness and major cations (Ca, K, Mg, Na) in all 47 prefectures in Japan.

Prefecture/Place	Hardness ($CaCO_3$ mg/L)					n	Ca (Mean±SD mg/L)	K (Mean±SD mg/L)	Mg (Mean±SD mg/L)	Na (Mean±SD mg/L)
	Min	Max	Median	Mean	SD					
Hokkaido	13.1	105	29.9	36.2	19.8	46	9.71±4.62	1.41±1.08	2.91±2.63	9.43±6.29
Aomori	8.41	46.7	22.6	26.7	12.0	11	7.03±4.24	1.47±0.60	2.22±0.74	14.7±7.81
Iwate	15.2	118	63.3	58.9	29.1	15	17.0±7.4	1.23±0.78	4.00±3.61	8.15±5.92
Miyagi	25.2	55.0	28.8	32.0	9.4	9	10.1±3.1	1.01±0.52	1.64±0.64	6.59±2.07
Akita	21.5	44.5	30.3	31.7	8.2	4	9.09±3.32	1.07±0.47	2.19±0.51	8.62±1.22
Yamagata	18.9	33.2	25.6	25.5	4.4	11	7.75±1.43	0.81±0.23	1.50±0.39	8.94±3.07
Fukushima	5.84	68.7	29.2	31.0	17.0	25	9.61±5.38	0.99±0.50	1.70±1.03	4.85±1.75
Ibaraki	14.2	90.9	57.9	55.8	20.4	29	15.1±5.0	3.38±2.15	4.38±2.42	14.4±8.2
Tochigi	27.3	94.6	60.3	63.7	21.9	8	18.0±5.9	1.78±0.46	4.55±2.64	9.40±4.27
Gunma	7.38	132	33.7	49.6	38.5	12	15.1±13.1	1.21±0.72	2.88±2.15	5.88±4.96
Saitama	44.5	200	79.3	82.8	31.1	17	24.1±8.8	2.91±0.93	5.50±2.65	14.7±5.5
Chiba	48.1	124	86.5	83.4	14.8	26	21.8±3.6	3.59±0.71	7.01±1.91	19.9±5.5
Tokyo	28.0	83.9	70.5	65.8	16.3	22	20.2±4.3	2.40±0.95	3.72±1.45	12.4±4.2
Kanagawa	1.16	98.2	54.6	56.2	18.5	33	15.9±4.9	1.06±0.48	4.15±1.60	7.48±5.11
Niigata	7.62	120	40.7	42.7	27.7	17	12.0±6.8	1.43±0.95	3.09±3.12	8.50±3.29
Toyama	17.3	75.6	30.6	35.6	15.9	16	12.0±5.7	0.81±0.44	1.40±0.79	7.67±8.95
Ishikawa	19.0	109	44.4	49.5	25.4	10	15.5±9.8	1.37±1.60	2.64±1.23	12.9±16.4
Fukui	19.5	55.9	34.5	35.3	13.0	7	10.3±3.8	1.08±0.66	2.31±1.63	9.92±6.82
Yamanashi	13.9	107	44.4	45.7	24.6	24	13.5±8.5	1.37±0.69	2.90±2.19	8.05±8.28
Nagano	14.8	152	46.8	57.0	36.8	24	14.9±7.6	2.16±1.26	4.82±4.97	9.68±8.15
Gifu	6.76	62.2	29.1	30.2	18.1	8	10.1±6.4	0.81±0.56	1.21±0.97	3.35±1.65
Shizuoka	17.4	92.5	47.1	50.3	16.5	21	15.0±5.4	1.23±0.52	3.13±1.29	6.27±2.55
Aichi	0.464	91.4	27.9	33.7	26.3	15	9.39±6.38	1.21±0.65	2.86±3.07	8.44±5.15
Mie	42.1	88.5	47.6	57.2	17.6	6	18.0±7.7	1.61±0.76	2.98±1.28	7.93±2.24
Shiga	24.7	52.3	42.2	41.6	7.3	9	13.6±2.4	1.41±0.51	1.87±0.47	7.92±3.30
Kyoto	25.5	56.3	46.4	45.3	9.2	11	13.4±3.8	1.76±0.72	2.89±0.83	10.9±3.2
Osaka	40.8	53.2	50.6	48.6	4.2	7	15.3±1.9	2.73±0.63	2.51±0.35	15.4±4.1
Hyogo	10.5	71.1	51.3	46.6	15.2	14	15.6±5.7	1.93±1.09	1.88±0.71	11.8±5.0
Nara	18.0	30.9	23.4	23.9	5.2	4	7.69±2.14	1.13±0.79	1.15±0.19	4.97±0.67
Wakayama	21.1	72.8	55.4	48.2	18.7	5	12.5±7.9	1.31±0.55	4.10±1.82	11.8±6.2
Tottori	20.5	106	35.5	43.3	18.8	32	11.4±4.2	1.92±0.95	3.59±3.09	12.0±5.5
Shimane	20.3	74.7	27.4	35.5	18.0	12	10.5±6.2	1.22±0.58	2.27±0.99	9.31±3.18
Okayama	12.2	105	42.9	45.4	21.5	29	14.8±7.9	1.29±0.56	2.05±0.90	13.4±31.3
Hiroshima	13.0	40.2	17.4	23.5	11.9	3	7.74±5.29	1.20±0.75	1.01±0.33	7.35±3.16

Yamaguchi	7.56	83.1	48.5	46.9	26.3	12	15.3±9.1	1.20±0.92	2.14±1.39	8.33±5.08
Tokushima	60.7	63.1	61.9	61.9	1.2	2	21.0±0.4	0.97±0.25	2.33±0.63	5.04±1.36
Kagawa	54.0	67.2	59.1	60.1	5.4	3	20.6±2.0	2.54±1.10	2.12±0.50	10.1±1.6
Ehime	33.8	56.9	52.2	47.6	10.0	3	15.5±4.2	1.17±0.19	2.19±0.90	6.40±1.62
Kochi	19.5	76.8	47.0	45.9	19.5	7	14.9±7.1	1.16±0.81	2.08±1.01	5.15±1.65
Fukuoka	1.41	77.4	47.8	47.5	15.6	17	14.9±5.6	2.15±1.13	2.51±1.16	12.0±8.4
Saga	14.4	53.1	34.1	33.9	14.1	4	10.2±5.0	1.92±1.39	2.04±1.00	10.0±3.2
Nagasaki	0.400	87.0	45.2	44.5	23.5	8	12.0±7.2	1.09±0.76	4.03±1.47	8.58±5.48
Kumamoto	39.8	101	75.6	72.2	19.2	10	19.0±6.0	4.40±2.44	5.99±2.18	14.5±6.7
Oita	18.5	108	55.9	57.9	25.2	16	14.5±6.2	3.24±1.54	5.27±3.15	13.8±7.0
Miyazaki	26.2	45.7	31.5	33.7	7.2	4	9.87±2.36	1.82±0.91	2.20±0.70	4.74±1.99
Kagoshima	20.9	113	39.1	45.7	21.8	17	12.9±7.5	2.43±1.45	3.27±1.47	15.7±12.1
Okinawa	0.101	186	65.4	68.1	38.6	16	20.8±14.3	1.84±2.27	4.16±1.34	22.0±11.5
Train	54.5	84.7	61.5	65.5	12.0	4	19.8±5.0	2.87±1.19	3.92±0.58	12.2±3.5
All	0.101	200	46.0	48.9	25.8	665	14.2±7.4	1.79±1.32	3.28±2.47	10.7±9.7

SD: Standard deviation (1σ), Min: minimum, Max: maximum, n: number of points

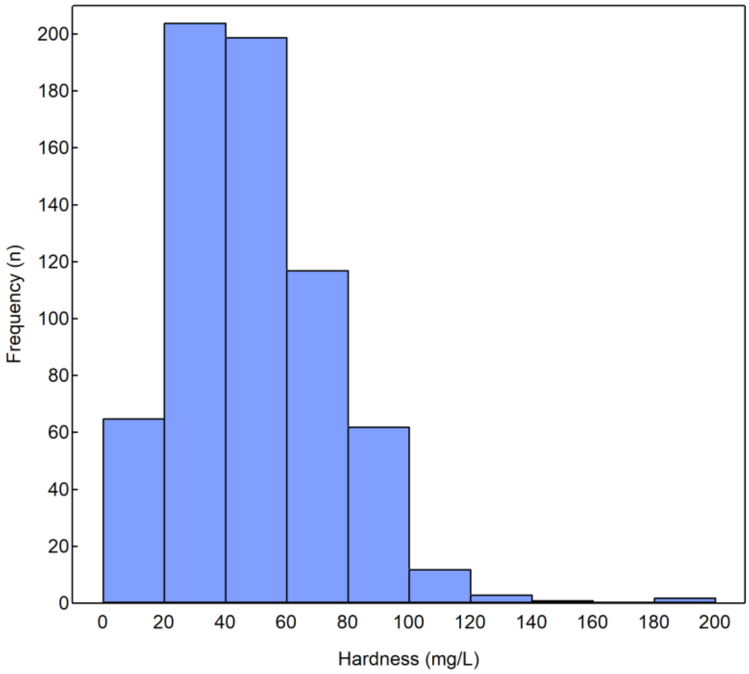

Figure S1. Histogram of the sampled tap water hardness in Japan (n = 665).

References

1. Ethiopian Standards Agency. Drinking water - specifications, Ethiopian standard CES 58, 2013. https://www.humanitarianresponse.info/sites/www.humanitarianresponse.info/files/documents/files/drinking_water_specifications.pdf (accessed 1 June, 2021)
2. Rural Drinking Water Quality Management Framework, Appendix 1: RWANDA STANDARD RS EAS 12: 2014, Second edition, Published by RSB 2014-11-28, Potable water – Speciation. https://www.mininfra.gov.rw/fileadmin/user_upload/Mininfra/Documents/Water_and_Sanitation_docs/2_Rural_Drinking_Water_Quality_Framework.pdf (accessed 1 June, 2021)
3. Zambia Bureau of standards. Zambian Standard, DRINKING WATER QUALITY - Specification, 2010. https://ec.europa.eu/growth/tools-databases/tbt/en/search/?tbtaction=get.project&Country_ID=ZMB&num=48&dspLang=en&basdatedeb=01/01/2016&basdatefin=31/01/2016&baspays=&baspays2=&basnotifnum=&basnotifnum2=&bastypepays=ANY&baskeywords=&project_type_num=1&project_type_id=1&lang_id=EN (accessed 1 June, 2021)
4. U. S. Environmental Protection Agency. National Primary Drinking Water Regulations. https://www.epa.gov/ground-water-and-drinking-water/national-primary-drinking-water-regulations (accessed 1 June, 2021)
5. Ministry of Health of China, National Standard of the People’s Republic of China GB 5749-2006 Standards for Drinking Water Quality, China, 2006. https://www.aqsiq.net/pdf/China_GB_5749-2006_Standards_for_Drinking_Water_Quality.pdf (accessed 1 June, 2021)
6. Ministry of Health, Labour and Welfare, Japan. Drinking Water Quality Standards. https://www.mhlw.go.jp/english/policy/health/water_supply/dl/4a.pdf (accessed November 20, 2020)
7. Ministry of Justice of the Republic of Kazakhstan, Approval of Sanitary Regulations "Sanitary and Epidemiological Requirements for Water Sources, Household and Drinking Intakes, Household and Drinking Water Supply, Cultural and Household Water Use Places, and Water Safety", 2015. (In Russian) http://adilet.zan.kz/rus/docs/V1500010774 (accessed 1 June, 2021)
8. The Regional Environmental Center for Central Asia. Water quality standards and norms in Kyrgyz Republic, 2009. http://www.cawater-info.net/water_quality_in_ca/files/kyrgyzstan_en.pdf (accessed 1 June, 2021)
9. Water Quality in Central Asia. http://www.cawater-info.net/water_quality_in_ca/standards_e.htm (accessed 1 June, 2021)
10. Ministry of Justice of the Russian Federation. Drinking water. Hygienic requirements for water quality in centralized drinking water supply systems. Quality control. https://ec.europa.eu/food/sites/food/files/safety/docs/ia_eu-ru_sps-req_sanpin_2-1-4-1074-01_20100628_en.pdf (accessed 1 June, 2021)
11. Government of Nepal, National Planning Commission Secretariat, Central Bureau of Statistics. Environment Statistics of Nepal 2019. https://unstats.un.org/unsd/environment/Compendia/Nepal_Environment%20Statistics%20of%20Nepal_2019.pdf (accessed 1 June, 2021)

12. Philippine National Standards for Drinking Water of 2017. http://water.emb.gov.ph/wp-content/uploads/2016/07/DAO-1994-26A.pdf (accessed 1 June, 2021)
13. Minister for the Environment and Water Resources, Singapore. Environmental Public Health (Water Suitable for Drinking) (No.2) Regulations 2019. https://sso.agc.gov.sg/SL-Supp/S274-2019/Published/20190401?DocDate=20190401 (accessed 1 June, 2021)
14. Environmental Protection Administration, Taiwan. Drinking Water Quality Standards, Taiwan, 2017-01-10. https://law.moj.gov.tw/ENG/LawClass/LawAll.aspx?pcode=O0040019 (accessed 1 June, 2021)
15. Drinking Water Quality Standard in Thailand. http://www.wepa-db.net/policies/law/thailand/std_drinking.htm (accessed 1 June, 2021)
16. Government of Abu Dhabi. The Water Quality Regulations (Fourth Edition). https://jawdah.qcc.abudhabi.ae/en/Registration/QCCServices/Services/STD/ISGL/ISGL-LIST/WA-705.pdf (accessed 1 June, 2021)
17. RSB Dubai. Water Quality Regulations. https://rsbdubai.gov.ae/wp-content/uploads/2020/07/RSB-Water-Quality-Regulations-1.0-1.pdf (accessed 1 June, 2021)
18. The Council of the European Union. COUNCIL DIRECTIVE 98/83/EC of 3 November 1998, on the quality of water intended for human consumption. https://eur-lex.europa.eu/legal-content/EN/TXT/?uri=celex%3A31998L0083 (accessed 1 June, 2021)
19. The United Nations Economic Commission for Europe (UNECE). Revised national targets of Armenia in the context of the UNECE-WHO-EUROPE protocol on water and health, Article 6, 2(a) Quality of the drinking water supplied, 2019. http://awhhe.am/wp-content/uploads/2019/01/EUWI-_Revised-National-Targets-Eng.pdf (accessed 1 June, 2021)
20. The Quality of Zurich's Drinking Water. https://www.stadt-zuerich.ch/content/dam/stzh/dib/Deutsch/BILDERwasserversorgung/3-Formulare_Merkblaeter/Trinkwasserqualit%C3%A4t%20englisch.pdf (accessed 1 June, 2021)
21. Ministry of Health, New Zealand. Drinking-water Standards for New Zealand 2005 (Revised 2018). https://www.health.govt.nz/publication/drinking-water-standards-new-zealand-2005-revised-2018 (accessed 1 June, 2021)
